# Serum Glucose and Malondialdehyde Levels in Alloxan Induced Diabetic Rats Supplemented with Methanolic Extract of Tacazzea Apiculata

**Published:** 2014-12

**Authors:** M. Y. Gwarzo, J. H. Ahmadu, M. B. Ahmad, A. U. A. Dikko

**Affiliations:** 1 Department of Medical Laboratory Science, Bayero University Kano, PMB 3011, Kano, Nigeria;; 2 National Biotechnology Development Agency, PMB 5118, Wuse-Abuja, Nigeria;; 3 Department of Human Physiology Faculty of Medicine, Bayero University k Kano PMB 3011 Kano, Nigeria

**Keywords:** Diabetes mellitus, Tacazzea apiculata, Oxidative stress, Antioxidant, Phytochemicals

## Abstract

*Tacazzea apiculata* is used by traditional medical practitioners for the treatment of wide range of diseases. The current work investigated the hypoglycemic and antioxidant properties of *Tacazzea apiculata* Oliv. on alloxan induced diabetes mellitus. Five groups (n=10) of rats were fed on commercial diet. The rats were divided into Group 1 (NUT) as non-diabetic and untreated, group 2 (NDT) as non-diabetic and treated, group 3 (DT) diabetic and treated. Group 4 (DUT) as diabetic and untreated. Group five (CP) were diabetic treated with Chlorpropamide, a drug used in the management of diabetic mellitus, with no known antioxidant property. Diabetic induction was done by intra-peritoneal injection of 100 mg/kg b. wt with alloxan. Fasting blood glucose was estimated seven days after induction to determine the severity of glucose elevation among the induced groups. Methanolic extract of T. *apiculata* leaf was administered to alloxan induced diabetic and non-diabetic control rats at 100mg/kg body weight for four weeks and blood glucose estimated on weekly basis. Malondialdehyde level was also estimated in the sera of the rats. Blood glucose level was monitored for additional 2 weeks post treatment. The results indicated that the extracts possess significant hypoglycemic effect on the diabetic rats (DT) having the mean glucose of (95.2 ± 9.12 mg/dl) compared to the diabetic untreated control group (DUT) with a mean glucose of (238.91 ± 4.42 mg/dl, *p*<0.05). The effect was sustained even on withdrawal of the extracts for two weeks. This was accompanied by a progressive increase in weight among all treated diabetic rats and non diabetic treated (DT and NDT) compared with diabetic untreated control rat (DUT) (*p*<0.05). A raised level in malondialdehyde was also observed among the diabetic rat prior to treatment and significantly decreased after the treatment. In conclusion the research demonstrated the hypoglycaemic and antioxidant potential of methanolic leaf extract of T. apiculata in alloxan induced rats.

## INTRODUCTION

Diabetes mellitus is a metabolic disorder characterized by hyperglycemia due to insufficiency of secretion or action of endogenous insulin. The high blood sugar produces the classical symptoms of polyuria (frequent urination), polydypsia (increased thirst) and polyphagia (increased hunger). Although the etiology of the disease is not well defined; viral infection, autoimmune disease, and environmental factors have been implicated (19, 35). Diabetic patients have significant defects of antioxidant protection which may increase their vulnerability to oxidative damage and the development of diabetes complications (9). Several lines of evidence show oxidative stress as a putative factor in the aetiology of many human disorders. Oxidative stress arises when reactive oxygen species (ROS) generation exceeds the available antioxidant defenses. ROS are chemically reactive molecules, containing oxygen e.g H_2_O_2_, HOCl, and free radicals such as superoxide anion, hydroxyl radicals (5). Antioxidants are molecules involve in scavenging of free radicals, this defence mechanism involve both enzymatic and non enzymatic strategies. Enzymes include superoxide dismutase (SOD), catalase, glutathione peroxidase and while enzymatic include small molecules include uric acid, vitamins E, C and A (5).

Malondialdehyde (MDA) is a degradative product of peroxidation of polyunsaturated fatty acids (PUFA) in the cell membrane, culminating in the generation of oxidative stress indicators, such as hydroperoxides (4). Reactive oxygen species occur naturally, but their accumulation is a marker for oxidative stress (22).

Medicinal plants are of great importance to the health of individuals and communities. Their medicinal values lie in some chemical substances they contain which form the basis of many of the modern pharmaceuticals. Medicinal plants represent an important health and economic component of biodiversity and also conservation and sustainable use. Information on the traditional knowledge of medicinal plants and their use represent a vital role in the discovery of novel products from plants as chemotherapeutic agents (6). In developing countries remedies from plant play an important role in the health care of millions of people. Despite immense technological advancement in modern medicine, many people in developing countries still depend on medicinal plants for their daily health care needs (23).

*Tacazzea apiculata* Oliv is woody climber indigenous to tropical Africa. It is popularly called Craw-craw vine. The Hausa of northern Nigeria refer to it as “*Yaadiyar kada*”. The powdered leaves are used in snake bite and stings by venomous animals. T. *apiculata* is also reported to be used in Hausa traditional medicine for the treatment of inflammation .The decoction of the root is used for convulsion and epilepsy by the Hausa people of North-western Nigeria (3). Analgesic and anticonvulsant activity of the methanol extract have been demonstrated (3). T. *apiculata* is also reported to be used in Hausa traditional medicine for the treatment of Diabetes (Personal communication).

Diabetes is probably the fastest growing metabolic disease in the world and knowledge of the heterogeneous nature of the disease increases so does the need for more alternative and appropriate therapies that would address the oxidative stress induced complication associated with the disorder. For centuries traditional plant remedies have been used for treatment of diabetes but only a few have been scientifically evaluated (15).

The aim of this research is to study the antidiabetic and antioxidant activity of *Tacazzea apiculata* plant extracts, in order to exploit its potential in the management of the disorder as well as the attending consequence of complication arising from oxidative stress.

## MATERIAL AND METHODS

### Plant Collection

The whole plant of *Tacazzea apiculata* were collected from a natural population in the new campus of Bayero University, Kano, and also at the forest of Falgore, Kano State, Nigeria and the plant was authenticated by Plant Science, Bayero University Kano, Nigeria.

### Plant Extract Preparation and Administration

The plant leaves were air dried under a shade and pounded into powder. Two hundred and fifty gram (250 g) of the powdered leaf was weighted into containers containing 2500 cm^3^ methanol and allowed to stay for four days with gentle shaking. The methanolic extract was evaporated using a rotary evaporator to obtain a solid extract of the plant leaf. Ten gram (10 g) of the solid methanolic extracts was dissolved in 100 cm^3^ of distilled water for use in the treatment of the experimental animals. The animals were given orally 100 mg/kg body of the methanolic extract dissolved in distilled water.

### Induction of Diabetes

The animals were fed on normal diet for 7 days of acclimatization. Diabetes was induced by a single dose of 100mg/kg body weight of alloxan monohydrate in freshly prepared 10 mmol/L sodium citrate, pH 4.5, intraperitoneally (IP), to rats fasting for at least 10 hours. Blood glucose levels were measured 3 days prior to induction and after 7 days of induction. Development of diabetes mellitus was proven by sustained hyperglycemia (>11.11mmol/L).

### Experimental Animals

A total of 50 Wistar (albino) rats weighing 120-200 g were bought from the animal house of Biological Sciences Department, Bayero University Kano. The animals were kept for the period of the study. They were housed in cages and kept in a room where a twelve hour light/dark cycle was maintained with free access to fed and water for a one week period of acclimatization before commencement of the experiment. The animals were fed on a commercially prepared growers mash obtained from PS Mandrides Plc, Kano, Nigeria.

### Experimental design

The rats were grouped into 5, with ten rats in each group. Each of the rats in a group was weighed after the grouping.

Group DT (diabetic and treated) Alloxan induced diabetic rats and treated with 100 mg/kg body weight (bw) of methanolic leaf extract of T. *apiculata*.

Group DUT (diabetic rats and un-treated) induced diabetic rats but not treated.

Group NDT (non-diabetic and treated) normal rats but treated with 100 mg/kg bw of leaf aqueous extract.

Group NUT (non diabetic and untreated rats (negative control).

Group (CT) Diabetic treated with Chlorpropamide.

### Treatment of the experimental Animals

The rats were treated for four weeks with either methanolic plant leaf extracts or Chlorpropamide. After four weeks, the treatment was terminated and half of the rats sacrificed by surgical dislocation of the neck. Blood was collected into flouride tubes, and centrifuged at 10,000 rpm. The serum obtained was stored at -80°C until required. The other half were left for two weeks for further observations after which they were treated as above. During the entire 6 weeks of the experiment, the fasting blood glucose of the rats was taken at weekly interval alongside their weight. The malondialdehyde level was also assayed at the termination of the experiment.

### Biochemical analysis

**Determination of Plasma Malondialdehyde (MDA).** Plasma malondialdehyde was measured by the method of Ohakawa *et al*. (22). Lipid peroxidation generates peroxide intermediates which upon cleavage release MDA, a product which reacts with Thiobarbitutic Acid (TBA). The product of the reaction is a coloured complex, which absorbs light at 532nm

**Methods.** 0.20 cm^3^ plasma was put in a test tube containing 3.0 cm^3^ of glacial acetic acid to which 3.0 cm^3^ of1% TBA in 2% NaOH was added. The mixture was placed in boiling water for 15 min. Absorbance of the pink coloured product was read at 532 nm after cooling. Calibration curve was constructed using malondialdehydetetrabutylammonium salt obtained from Sigma (St Louis USA).

**Determination of Blood Glucose Level.** Blood samples of the rats were collected by cutting the tail tip of the rats for blood glucose determination before administering the extract. Administration of the extract or Chlorpropamide commenced 7 days after induction for a period of 28 days. Blood glucose level was determined based on Glucose Oxidase Method (7) and results were reported as mg/dL.

**Phytochemical Sreening.** The phytochemical screening of the crude extract of *Tacazzea apiculata* was done using the standard methods of sofowora (29), Trease and Evans (31) and El Olemy *et al.* (12). The phytochemicals secreened using these methods include phenolics glycosides, tannins, flavonoids, proanthocyanidins, alkaloids, anthraquinones and saponins.

**Determination of Total Flavonoids, Total Proanthocyanidins and Total Phenolics.** Method of Ordonez *et al*. (24) was used to determine total flavonoids using quercetin as standard. Whileproanthocyanidins was estimated by the method of Sun *et al.* (30). Total phenolic compounds were determined by Folin Ciocalteu method as adopted by Wolfe *et al*. (36). For both total phenolic acids and proanthocyanidins, tannic acid was used as standard.

### Statistical analysis of data

The mean and standard error of means were obtained for all data analysis using spss4 programme. Instat 3 was used for the analysis of variance (anova) to determine the level of significant. P values less than 0.05 (*p*<0.05) were considered significant.

## RESULTS

### Mean serum glucose level during extract administration

Table 1 shows the result of fasting serum glucose levels. The results were of mean+STD. There was a significant increase in fasting serum glucose level alloxan induced diabetic rats (*p*<0.05) compared with uninduced group (NUT) before dietary supplementation. While significant decrease in fasting serum glucose level (mg/dl) in the rats from the first to the fourth week treated with supplement and Chlorpropamide was observed. A gradual reduction of serum glucose level but not significant was also observed in non diabetic supplemented group (NDT) compared with uninduced group on unsupplemented diet (NUT).

**Table 1:** Fasting Blood Sugar of Alloxan Induced Diabetic Rats Treated with T. *Apiculata* Leave Extracts

	GROUPS	INDUCTION	WEEK1	WEEK 2	WEEK 3	WEEK 4

LEAF	DT	251.00 ± 22.75^a^	96.80 ± 8.42^b^	93.20 ± 8.56^b^	95.60 ± 10.38^b^	86.42 ± 2.98^b^
	NDT	98.20 ± 1.77^b^	71.20 ± 1.32^b^	71.20 ± 1.32^b^	69.40 ± 0.93^b^	70.00 ± 3.36^b^
	DUT	231.32 ± 6.49^a^	248.14 ± 5.61^a^	236.12 ± 2.92^a^	237.16 ± 5.83^a^	234.23 ± 3.31^a^
	NUT	110.80 ± 4.02^b^	102.90 ± 2.49^b^	98.60 ± 5.81^b^	96.20 ± 1.46^b^	87.20 ± 2.52^b^
CONTROL CP (84mg/kg)		232.21 ± 3.55^a^	120.00 ± 1.36^b^	121.78 ± 1.54^b^	98.88 ± 2.33^b^	99.12 ± 1.22^b^

DT, diabetic treated; NDT, non-diabetic treated; DUT, diabetic untreated; NUT, non-diabetic untreated Values are mean ± standard error of mean (n=4); CP, Chlorpropamide.

a Statistically different (*p*<0.05) Compared with NUT;

b Statistically different (*p*<0.05) compared with DUT.

Table 2 shows the fasting serum levels two weeks of withdrawal of dietary supplementation. There was a slight increase but not significant in the mean serum glucose level (mg/dl) in the second week after withdrawal plant extract supplementation in diabetic treated (DT) compared with non-diabetic control (NDT). The fasting serum glucose levels two weeks after withdrawal of dietary supplement in diabetic treated (DT), non-diabetic treated and non-diabetic control (NUT) were significantly (*p*<0.05) lower than the diabetic control (DUT). The level of serum glucose two weeks after diet supplementation was normal in contrast to the group treated with chlorpropamide (CP) in which there was a gradual and significant increase in fasting serum glucose level after withdrawal of treatment (*p*<0.05).

**Table 2:** Fasting Blood Glucose (Mg/Dl) Levels after Withdrawal of Treatment with T.*Apiculata* Leaf Extracts

	Group	Week 1 Glucose (mg/dL)	Week 2 Glucose (mg/dL)

**Treated**	**DT**	83.60 ± 10.99^a^	105.20 ± 5.74^a^
	**NDT**	84.00 ± 1.92^a^	92.20 ± 1.36^a^
	**CP**	130 + 0.50^a^	180 ± 1.23^a^
**Control**	**DUT**	234.11 ± 13.19	222.32 ± 11.66
	**NUT**	93.40 ± 3.59^a^	107.90 ± 2.49^a^

DT, diabetic treated; NDT, non-diabetic treated; DUT, diabetic untreated; NUT, non-diabetic untreated; CP, chlorpropamide. Values are mean ± standard error of mean (n=5).

a Statistically significant (*p*<0.05) compared with diabetic control (DUT).

Malonialdehyde serum concentration in the experimental animals is shown in Table 3. Serum malonialdehyde was significantly lower in Diabetic dietary supplemented (DT) group (*p*<0.05) compared with Diabetic untreated control (DUT) group. Supplementation reduced the level of serum malonidaldehyde in non diabetic treated group (7.2 ± 0.54 mmol/L) compared with non-diabetic untreated control group (8.5 ± 0.76 mmol/L). The Level of serum malonialdehyde level in diabetic treated was not significantly different from diabetic group on Chlorpropamide.

**Table 3:** Malonyldialdehyde Concentrations in Alloxan Induced Diabetic Rats and Controls after 28 Days of Treatment

GROUPS	MALONDIALDEHYDE (mmol/L)

DT	9.5 ± 0.86^a^
NDT	7.2 ± 0.54^a^
DUT	18.3 ± 1.26
NUT	8.5 ± 0.76^a^
CP	9.2 ± 0.76^a^

DT, diabetic treated; NDT, non-diabetic treated; DUT, diabetic untreated; NUT, non-diabetic untreated; CP, Chlorpropamide. Values are mean ± standard error of mean (n=5).

a Statistically significant (*p*<0.05) compared with diabetic control (DUT).

### Phytochemical Sreening

The phytochemical screening of the crude extract of Tacazzea apiculatawas carried out in order to ascertain the presence of its constituents utilizing standard methods Sofowora (24). The analysis showed the presence of alkaloids, flavanoids, proanthocyanidins and Tannins among other classes of phytochemicals as shown in Table 4. Anthroquinones were the only group of phytochemicals not detected in the extract.

**Table 4:** Phytochemical constituents present in extract of Tacazzea apiculata leaves

Constituents	Inference

**Alkaloids**	+
**Glycosides**	+
**Flavonoids**	+
**Proanthocyanidins**	+
**Tannins**	+
**Saponins**	+
**Terpenoids**	+
**Steroids**	+
**Anthraquinones**	+

+, means present.

### Polyphenolic Contents methanolic Tacazzea apiculata leaf extract

The polyphenolic quantitative analysis indicated that Total phenolic acid compounds has the highest content (22.12 ± 2.11 mg/g weight of the extract.), followed by flavanoids with content concentration of 18.23 ± 0.75 mg/g weight of the extract. The content of proantocyanidins was the least in concentration (11.50 ± 1.02 mg/g weight of the extract compared with total phenolics and total flavonoids (Table 5).

**Table 5:** Concentration of polyphenolic of methanolic Tacazzea apiculata leaf extract

Constituents	Concentration (mg/g extract)

Proanthocyanidins	11.50 + 1.02
Total phenolics	22.12 + 2.11
Total Flavonoids	18.23 + 0.75

Proanthocyanidins and Total phenolics are expressed as mg tannic acid/g dried leaf methanolic extract while total flavanoids is expressed as mg quercetin/g dried leaf methanolic extract.

## DISCUSSION

Since the understanding that the selective destruction by alloxan of pancreatic beta cells is mediated via generation of oxidative stress (15), effort has been targeted towards the use of anti-oxidant to prevent chemically induced damage of pancreatic beta cells. Toxicity of alloxan is elicited through its reduction by glutathione to dialuric acid in which redox recycling process generates ROS that damages the beta cells (15). Malondialdehyde (MDA) is degradative product of peroxidation of polyunsaturated fatty acids (PUPA) in the cells membrane. Presence of higher MDA in the serum is an indication of induced lipid peroxidation and of oxidative stress of which has been reported as one of the underlying cause of diabetes mellitus (4, 9). Although the concentration of the MDA across the set groups were not significantly higher compared to that of the control non diabetic group, however, the values obtained from the diabetic treated (DT) were all high compared to that of the control groups (NUT,NDT).

Diabetic group (DT) on plant supplement was normoglycaemic during the course of the experiment. This may be related to the effect of some elemental constituents such as Zinc, Copper, Magnesium, manganese that function in part as an antioxidants (3, 12, 34), and the presence of flavonoids and proanthocyanidins as demonstrated by phytochemical in this study. The phytochemical analysis confirmed the presence of constituents which are known to exhibit medicinal as well as physiological activity. The detection of flavonoids in the extract can be linked to the blood glucose lowering property. Flavonoids inhibit glucose-6-phophatase activity in the liver thereby suppressing gluconeogenesis and glycogenolysis and consequently reduces the hyperglycaemia. (10). Polymers and oligomers from proanthocyanidins of Persimmon inhibit digestive enzymes such as amylase and glucosidase enzymes in addition to prevention of the formation of advanced glycation products (17). Hence, it is likely that the proanthocyanidins in the leaves may have as well reduce the glycaemic index of the food consumed by acting on the carbohydrate digestive enzymes such as amylase and glucosidase. Furthermore, Several flavanoids were described as hypoglycemic agents (16). Tacazzea apiculata Oliv. is widely distributed in tropical Africa and the parts are considered medicinal (1, 8, 25). It is shown to contain polyphenols in this study. Bioavalibility of polyphenols is known to be affected by glycosidic conjugation (16). Intestinal sodium glucose transporter-1 (SGLT-1) was suggested to be involved in the absorption of quercetin glucosides (37). Hence they competitively inhibit sodium (Na+) dependent mucosal uptake of the non-metabolisable glucose analogue methyl-α-D-glucopyranoside via SGLT-1 using rat mid-jejunum, whereas quercetin (aglycone) and rutin had no effect (2). Similarly, conjugated flavonoids such as Quercetin-3-glycosides have the tendency of inhibiting Na+-independent non-saturable uptake of glucose by SGLT-1 (2). *Tacazzea apiculata* is reported to contain trace elements (3). Some of these elements include Mn, Cu, Zn, Se and Fe are known to act as cofactors for endogenous antioxidants such as glutathione peroxidase (GSHPx), catalase (CAT) and superoxide dismutase (SOD) for optimum catalytic activity and effective antioxidant defense mechanism against oxidative stress (28). Therefore, the ability to attenuate oxidative stress in diabetic group treated with supplement might be a consequence of the presence of these trace elements synergized by the possible transcriptional activation of the endogenous antioxidants proteins by polyphenolic compounds (20) whose catalytic activities are potentiated by the trace elements.

Rajendran *et al*. (26) reported the wide range of medicinal applications of Aloe Vera, which include hypoglycaemic effect in diabetes, ulcer curative effect, stimulating immune response against cancer. *Sclerocarya birrea was* also reported to have evaluated for anti-inflammatory and anti-diabetic properties in animal experiments (23). These effects are being attributed to the role of inorganic elements like zinc, chromium, vanadium, iron, copper and manganese in the improvement of impaired glucose tolerance. Thus, the indirect role of these elements in management of diabetes mellitus are being increasingly recognized (27). Keeping the above facts in view, Tacazzea apiculata was reported to have similar elemental composition to which the hypoglycemic effect could be related (3).

Magnesium is one of the major minerals involved in carbohydrate and fat metabolism. Hypomagnesaemia has been incriminated in the development and progression of diabetic retinopathy and defective release of insulin (27).

A correlation has been established between decrease in manganese level and severity of diabetes mellitus in experimental animals and its supplementation reversed the impaired glucose utilization induced by its deficiency in guinea pigs. Manganese also potentiates the action of insulin in animals with low insulin level, thereby increasing the transport of glucose into adipose tissue (27, 32).

Zinc modulates diabetic condition either by acting as a cofactor for insulin (11) or a component of cytosolic superoxide dismmuatse enzyme. Abnormal zinc metabolism has been suggested to play a role in the pathogenesis of diabetes and its complications due to diminution of its role in insulin action and antioxidant capacity of cytosolic superoxide dismutase (32, 34).

Normal potassium concentration is necessary for optimal insulin secretion while depletion can result in reduced glucose tolerance. Deficiencies arise in abnormal conditions such a diabetic acidosis (32).

Vanadium was reported to elicit glucose lowering and cardio-protective effect in streptozotocin induced diabetic rats. Numerous investigations have demonstrated the beneficial effect of vanadium salts on diabetes in STZ diabetic rats, in rodents with genetically determined diabetes and in human subjects. Vanadium was reported to mimic the metabolic effects of insulin in rat adipocytes. Subsequent studies revealed that vanadium therapy was shown to normalize blood glucose levels in streptozotocin diabetic induced rats (33). Interestingly, in this study, withdrawal of supplement did not resort gradual hyperglycaemia as noticed with in chlorpropamide treated diabetic rats. It is likely the the methanolic extract in addition to having hypoglycaemic effect may have as well the potential of regeneration the β cells destroyed by alloxan. Future work will attempt to determine such action as well as its potential to generation of β cells destroyed by autoimmune condition associated with type 1 diabetes mellitus in human.

Thus the presence of flavonoids, procynidines and essential trace elements as identified in this plant may readily account for the most of the therapeutic efficiencies. The identified above compounds may lay a direct or an indirect role in insulin secretion or action in a synergistic manner and therefore have a significant role in anti-diabetic activity.
